# Molecular sensitization clusters in adult atopy: an ALEX^2^-based analysis from Eastern Europe

**DOI:** 10.3389/fimmu.2026.1960324

**Published:** 2026-09-18

**Authors:** Alexandra Chera, Selda Ali, Courtney Chau, Benjamin Hu, Alanis Villanueva, Emma Guttman-Yassky, Octavian Bucur, Roxana-Silvia Bumbacea

**Affiliations:** 1 Carol Davila University of Medicine and Pharmacy, Bucharest, Romania; 2 Department of Allergy and Clinical Immunology, ‘Dr. Carol Davila’ Clinical Nephrology Hospital, Bucharest, Romania; 3 Genomics Research and Development Institute, Bucharest, Romania; 4 Department of Dermatology, Icahn School of Medicine at Mount Sinai, New York, NY, United States

**Keywords:** ALEX^2^ multiplex assay, allergic rhinitis, asthma, atopic dermatitis, cluster analysis

## Abstract

**Background:**

Allergen sensitization profiles vary geographically, yet Eastern Europe data remain scarce. This study aimed to identify and characterize molecular sensitization clusters in adults with allergic rhinitis (AR), asthma, and/or atopic dermatitis (AD) at a Romanian tertiary allergy center using ALEX^2^ multiplex profiling.

**Methods:**

Among 213 consecutive adults undergoing ALEX^2^ testing (January 2024–May 2026), 161 with confirmed AR, asthma, or AD formed the study population. Of these, 139 with at least one detectable allergen sensitization were included in Ward.D2 hierarchical clustering across 98 allergens. Associations were evaluated using Fisher’s exact test with Benjamini-Hochberg correction.

**Results:**

The present study identified four primary molecular allergen sensitization clusters in an adult atopic cohort from Romania, with an exploratory, five-cluster refinement further resolving heterogeneity within the dominant cluster. Cluster 1 (pauci-sensitized, n=42) paradoxically showed a high asthma prevalence (54.8%) despite the lowest sensitization breadth (median 2 sensitizations) and total IgE (133.5 kU/L), though this finding did not reach significance in the global cluster comparison and should be considered exploratory. Cluster 2 (grass pollen-dominant, n=24) and Cluster 3 (polysensitized, n=47) showed near-universal AR prevalence. Cluster 4 (multi-panallergen PR-10/nsLTP, n=10) carried the highest rate of oral allergy syndrome (OAS) (30%), driven by PR-10 and nsLTP, whereas Cluster 5 (ragweed and profilin-dominant, n=16) showed exclusively profilin-mediated OAS. Age was negatively correlated with sensitization breadth (Spearman r= -0.265, p=0.0016), with paradoxically elevated total IgE in older patients.

**Conclusion:**

Unsupervised clustering of ALEX^2^ data identified four primary molecular sensitization phenotypes in a Romanian adult atopic cohort, with an exploratory five-way refinement resolving additional clinically relevant heterogeneity, representing one of the first such characterizations from Eastern Europe. Key clinical insights include a pauci-sensitized cluster with an exploratory enrichment for asthma and a region-specific ragweed/profilin cluster, highlighting the utility of multiplex molecular profiling in adult atopic disease.

## 1 Introduction

Allergic rhinitis (AR), asthma, and atopic dermatitis (AD) are among the most prevalent chronic conditions worldwide (1). Distinct patterns of allergen sensitization, particularly the co-occurence and interactions between IgE responses to different allergens rather than sensitization alone, have been shown to predict the development, severity, and persistence of atopic diseases, with polysensitized profiles associated with more severe asthma phenotypes in some pediatric cohorts (2). However, the reported rates of IgE-mediated sensitization in atopic cohorts varies significantly: the MeDALL (Mechanisms of the Development of ALLergy) study demonstrated that although the co-occurrence of asthma, AR and AD is approximately 50% more common than expected by chance, allergen sensitization only accounted for 38% of the excess disease comorbidity (1, 3, 4), while allergen sensitization rates of up to 91% have been reported in hospitalized asthmatic children (5). These findings suggest that additional mechanisms extending beyond IgE-mediated pathways might contribute to shared atopic disease risk (6, 7). The strength of association between specific IgE (sIgE) values and atopic diseases has also been disputed over time, and it is hypothesized that IgE responses to multiple allergens are functionally coordinated and co-regulated, therefore the interaction patterns might foreshadow disease development (3, 7, 8).

Precision medicine has progressively adapted the landscape of allergy diagnosis, either through singleplex or multiplex allergen microarrays which are currently considered a standard complementary approach to *in vivo* tests, enhancing diagnostic specificity and offering valuable insights especially when skin testing is not feasible (9–11). Component-resolved diagnostics (CRD) enables detailed sensitization profiling, differentiation between primary co-sensitization and cross-reactivity driven by panallergens, and informed allergen immunotherapy selection (AIT) (8, 12). The Allergy Explorer (ALEX) test, one of the broadest molecular allergen multiplex platforms, became commercially available in 2018 and was able to simultaneously measure sIgE for 282 allergens. At the end of 2019 it was updated to ALEX^2^, measuring sIgE values for 295 allergen reagents, while in 2025 the ALEX^3^ version became available, testing 300 allergens in a single blood sample, including 218 allergen components and 82 extracts (11, 13). These CRD arrays generate rich datasets amenable to machine learning and network analysis. Unsupervised statistical learning techniques offer a data-driven approach to identifying latent sensitization subgroups from these rich datasets, without reliance on predetermined classifications; methods such as hierarchical clustering or latent class analyses (LCA) have been applied in order to identify atopic phenotypes. However, inconsistencies still remain and are largely attributable to differences in variable selection and study population characteristics (3, 9).

Sensitization profiles are heterogeneous between geographic regions due to the influence of local environmental exposures, ancestry, and socioeconomic factors, therefore, findings from one region may not apply to another (8, 14, 15). Anthropogenic changes to aeroallergen exposures, particularly pollen and fungal spores, further underscore the need for region-specific characterization (12).

Large multi-country European cohort studies, mostly involving Western and Central countries, have provided foundational insights into the relationship between sensitization patterns and atopic disease. Building on the MeDALL findings described above, unsupervised learning studies conducted on the MAS (Multicenter Allergy Study) and PASTURE (Protection against Allergy: Study in Rural Environments) cohorts have revealed that only excessive sIgE production early in life independently affected asthma risk (16). Across 11 European countries, the U-BIOPRED (Unbiased Biomarkers for the Prediction of Respiratory Disease Outcomes) consortium confirmed that asthma severity correlates with the strength of interactions between component-specific IgE responses, particularly for furry animal proteins, rather than the number of sensitizations (6).

Across Western Europe, France was one of the first countries to use unsupervised learning for characterizing asthma endotypes through cluster analyses in 2012 (17). More recently, the SAMP (Severe Asthma Molecular Phenotype) megacity cohort study from Paris identified sensitization to the tropomyosin molecular family as a novel finding associated with severe asthma (2), while the EGEA (Epidemiological study on the Genetics and Environment of Asthma) cohort identified four sensitization profiles which remained stable from childhood to adulthood, with 73% of participants retaining their sensitization profile over the age of 12 (18). In the COBRAPed (Cohort of Obstruction and Bronchial Recurrence in Asthma in Pediatrics) study, sensitization patterns did not discriminate between mild and severe wheeze, suggesting involvement of both IgE and non-IgE mechanisms in the development of asthma (19). In the United Kingdom, the Manchester Asthma and Allergy Study (MAAS) has provided many useful insights regarding associations between allergen sources and atopic outcomes (3), specific phenotypes correlated to clinical trajectories involving the development of atopic disease (e.g. the ‘complete mite sensitization’ was the only trajectory associated with comorbid asthma, AR, and AD) (20), and pairwise interactions between sIgE to different allergens emerged as asthma predictors (e.g. grass pollen/cat dander cluster was the strongest early life predictor of asthma at age 16) (7, 21). More recently, within-cluster sensitization breadth at age 5 was shown to predict the prevalence of asthma at age 16, with connectivity networks increasing progressively in complexity with age following a defined temporal hierarchy of allergen sensitization (8, 22).

In Northern and Central Europe, population-based studies from Finland and Estonia identified similar sensitization profiles correlated with markedly different socioeconomic backgrounds (15). In Denmark, seven distinct sensitization patterns were identified in young children, each associated with different atopic outcomes, and verified in an independent Swedish cohort (23). In Luxembourg, young adults showed the highest allergen sensitization burden, with tree pollens, grass, and house dust mites (HDM) as the predominant sources (24). In the Czech Republic, studies regarding the landscape of allergen sensitization have focused on AD, correlating storage mite NPC2 proteins, lipocalins, and PR-10 proteins with increased severity, while cluster analysis revealed cross-reactivity patterns relevant for patients allergic to crustaceans (9, 10).

In Southern Europe, oral allergy syndrome (OAS) in Italian children with seasonal AR was classified into five distinct endotypes based on panallergen sensitization, each with specific clinical presentations (25). In Portugal, five longitudinal sensitization profiles were identified from early childhood, with HDM-dominant trajectories associated with asthma and AR, while integration of CRD into predictive algorithms improved asthma prediction accuracy at age 13 (14, 26).

In Asia, HDM dominates as the leading sensitizing source across multiple countries, while pollen sensitization predominates in Western Chinese regions, and distinct sensitization classes with differing disease associations have been described in both China and Korea (27–29). In Turkey, hierarchical clustering revealed cross-reactivity patterns among 2S albumins, lipocalins and uteroglobins, with clinical relevance for allergy management (30, 31). In the United States, 91% of children hospitalized for asthma were sensitized to at least one allergen, with a high prevalence of polysensitizationand co-sensitization patterns more informative than aggregate counts (5). In South Africa, a coastal temperate sensitization pattern was confirmed with high grass pollen sensitization, particularly to Bermuda grass (12).

Populational studies regarding molecular sensitization profiles in Eastern Europe remain scarce. The most consistent and well-documented finding in this geographic area pertains to ragweed pollen sensitization, which has emerged as a dominant regional allergen due to the exceptionally high Ambrosia artemisiifolia pollen burden found in the Pannonian plain, with high sensitization rates reported in both Hungary (32) and Romania (33–35). Beyond ragweed, no comprehensive molecular sensitization analysis using a multiplex CRD platform has been conducted in Eastern European atopic populations. To our knowledge, the present study represents the first unsupervised learning analysis of molecular allergen sensitization profiles across atopic diseases in a Romanian adult cohort, using the ALEX^2^ multiplex platform.

## 2 Methods

### 2.1 Study population

We retrospectively analyzed a cohort of 213 patients who consecutively underwent ALEX^2^ multiplex testing at the Allergy and Clinical Immunology Department of our tertiary center in Bucharest, Romania, between January 2024 and May 2026. ALEX^2^ testing was indicated by the treating Allergy specialist as part of an extended allergological evaluation comprising clinical history, physical examination, and *in vivo* testing (skin prick tests), in cases where molecular sensitization profiling was deemed necessary to complete the diagnostic work-up. Diagnoses were established by an Allergy specialist based on the complete clinical and diagnostic evaluation and coded according to the International Classification of Diseases, 10th revision (ICD-10), ensuring standardized diagnostic categorization across the cohort. Diagnoses of AR were established based on clinical history, skin prick testing, and sIgE measurement in accordance with ARIA guidelines (36), asthma was diagnosed according to GINA criteria (37), and AD was diagnosed according to Hanifin and Rajka criteria (38). OAS was diagnosed clinically by an Allergy specialist, based on patient history, reported culprit foods, and reaction characteristics, prior to and independently of ALEX^2^ testing. The specific molecular subtype (PR-10-associated, profilin-associated, or nsLTP-associated) was subsequently determined by integrating this clinical information with ALEX2 molecular results, since the culprit protein family often cannot be reliably inferred from clinical history alone. Demographic data collected included age, sex, and residential environment (urban/rural). Clinical data comprised atopic disease diagnoses and the presence of OAS.

For inclusion in the present analysis, the patients had to be over 18 years old and were required to carry a confirmed diagnosis of at least one of the three major atopic diseases: AR, asthma, or AD.

Patients without any of the three atopic triad diagnoses after establishing the final diagnosis, namely patients suffering from non-allergic rhinitis, isolated IgE-mediated food allergy, hymenoptera venom allergy, or eosinophilic esophagitis were excluded from the final cohort (52 patients were excluded, n = 161).

For the hierarchical cluster analysis, 22 patients with no detectable allergen sensitization on ALEX^2^ testing were excluded, as their inclusion would not contribute meaningful signal to a sensitization-based clustering approach. These patients are present in the overall cohort description (in **Table 1**, we included a ‘Not sensitized’ row) but were excluded from all subsequent sensitization-based analyses. The final analytical cohort consisted of 139 patients.

**Table 1:** Demographic, clinical, and sensitization characteristics of the study population after applying the exclusion criteria (n = 161).

Characteristic	All patients (n = 161)	Allergic rhinitis (n = 135)	Asthma (n = 64)	Atopic dermatitis (n = 40)
Age (years)	35 (26, 48)	34 (25, 47.5)	42.5 (28, 55)	28.5 (23.8, 41.2)
Sex				
Female	103 (64%)	82 (60.7%)	48 (75%)	26 (65%)
Male	58 (36%)	53 (39.3%)	16 (25%)	14 (35%)
Rural/Urban				
Urban	134 (85.9%)	116 (87.9%)	56 (90.3%)	32 (80%)
Rural	22 (14.1%)	16 (12.1%)	6 (9.7%)	8 (20%)
Total IgE kU/L	203 (85.5, 1097)	223 (91, 995)	254 (88, 1237.5)	427 (85.5, 2314.5)
Sensitization status				
Sensitized (on ALEX2 test)	139 (86.3%)	122 (90.4%)	55 (85.9%)	33 (82.5%)
* Not sensitized (on ALEX2 test)*	*22 (13.7%)*	*13 (9.6%)*	*9 (14.1%)*	*7 (17.5%)*
Median number of sensitizations*	10 (4, 15.5)	10 (5, 16.8)	8 (2, 16)	12 (3, 16)
Atopic diseases				
Allergic Rhinitis	135 (83.9%)	135 (100%)	47 (73.4%)	29 (72.5%)
Asthma	64 (39.8%)	47 (34.8%)	64 (100%)	13 (32.5%)
Atopic Dermatitis	40 (24.8%)	29 (21.5%)	13 (20.3%)	40 (100%)
Oral allergy syndrome	11 (6.8%)	11 (8.1%)	4 (6.2%)	4 (10%)

Median (Q1, Q3); n (%). *Among sensitized patients only. 5 patients had missing Rural/Urban data and were excluded from that calculation. Patients may appear in more than one disease column due to overlapping diagnoses.

### 2.2 Molecular sensitization assessment

Allergen-specific IgE values were measured using the ALEX^2^ Allergy Explorer (MacroArray Diagnostics, Vienna, Austria) (11), an *in vitro* multiplex immunoassay based on a nanotechnology that enables simultaneous measurement of total IgE (tIgE) and sIgE against 295 allergen reagents, comprising 117 allergenic extracts and 178 molecular components, from 100 microliters of serum or plasma (10, 11, 13).

Sensitization to an allergen is defined by sIgE values ≥ 0.3 kU_A_/L.

### 2.3 Statistical analysis

Unsupervised hierarchical cluster analysis was performed to identify molecular sensitization patterns among atopic patients (diagnosed with AR, asthma and/or AD).

For the clustering analysis, we transformed the sIgE results obtained through the ALEX^2^ multiplex assay into binary code, with ‘1’ codifying sensitization (sIgE ≥ 0.3 kU_A_/L) and ‘0’ indicating sIgE values lower than 0.3 kU_A_/L. Only patients with at least one allergen sensitization were included in the clustering analysis (n = 139). Prior to cluster analysis, allergens were filtered based on their prevalence within the analytical cohort, and were retained if present in at least 4 of the 139 sensitized patients (n ≥ 4). This operational threshold derived from a norminal 3% prevalence criterioon (3% x 139 = 4.17, rounded down to the nearest integer). Allergenscausing sensitizations in fewer than 4 patients were excluded, as their inclusion would only introduce disproportionate noise to the clustering without providing meaningful discriminatory information. After filtering, 98 of 295 allergens were retained for analysis. The retained allergen extracts and molecular components are listed in full in **Supplementary Table S1**. This filtering approach is consistent with previously published multiplex-based clustering studies using the ALEX^2^ platform, including the SAMP cohort (2).

Euclidean distance was used as a dissimilarity measure and Ward’s minimum variance method (Ward.D2) as the linkage criterion, consistent with previously published ALEX^2^-based clustering studies (2). Ward’s method was selected because it minimizes within-cluster variance and tends to produce clinically interpretable, compact clusters. As binary Euclidean distance treats joint absences and joint presences symmetrically, we additionally performed a sensitivity analysis re-running the identical Ward.D2 clustering pipeline using Jaccard (binary) distance, which disregards joint negative results, and agreement between the two solutions was assessed using the adjusted Rand index (ARI). Although the minimum-variance objective of Ward’s method is derived for Euclidean space, coupling Ward.D2 linkage with Jaccard distance was employed strictly as a pragmatic sensitivity check to isolate the influence of joint absences on the cluster structure, while holding the linkage algorithm constant. Following the methodology of the MeDALL consortium (1), we did not perform dimensionality reduction to the input data prior to clustering, in order to preserve clinical interpretability. The optimal number of clusters was determined by combining visual inspection of the dendrogram with average silhouette width analysis tested across k = 2 to 8 (**Supplementary Figure S1**). Following the approach of previously published multiplex-based clustering studies (2, 3), the final number of clusters was selected based on both mathematical criteria and biological interpretability. Although the silhouette width was highest at k=4 (0.18), the k=5 solution (silhouette width = 0.08) was ultimately preferred, as the k=4 solution yielded a dominant cluster comprising 64% of patients that upon further examination contained two biologically and clinically distinct subgroups. To assess the robustness of the selected five-cluster solution relative to the mathematically optimal four-cluster solution, we performed a bootstrap stability analysis using the clusterboot function (fpc package, R), applying the same Ward.D2/Euclidean clustering pipeline to 1,000 bootstrap resamples of the analytical cohort and calculating the mean Jaccard similarity coefficient for each cluster.

Differences in continuous variables across clusters were assessed using the Kruskal-Wallis test. Categorical variables were compared across clusters using Fisher’s exact test with Monte Carlo simulation (B = 10,000 permutations), which was preferred over the chi-squared test given the small sample sizes in some clusters. For cluster-specific associations (one cluster versus all others), standard Fisher’s exact test was applied. A p-value < 0.05 was considered statistically significant throughout. All p-values for multiple comparisons were corrected using the Benjamini-Hochberg false discovery rate (FDR) method.

The sensitization prevalence of each of the 98 retained allergens was calculated within each cluster in order to provide molecular characterizations, and one-vs-rest Fisher’s exact tests were performed for each of the 490 allergen-cluster pairs (98 allergens × 5 clusters), with FDR correction applied as described above. Allergens with a FDR-adjusted p-value < 0.05 were considered significantly associated with a given cluster. To complement the hierarchical clustering and provide an independent visualization of the sensitization landscape, Uniform Manifold Approximation and Projection (UMAP) was performed on the binary sensitization matrix using the R package *umap* (n_neighbors = 15, min_dist = 0.1, Euclidean distance metric). UMAP was applied exclusively for *post-hoc* visualization and did not influence cluster assignments. UMAP was preferred over Principal Component Analysis (PCA) given the binary nature of the input data, as PCA assumes continuous normally distributed variables and may distort distances in binary datasets. A fixed random seed (set.seed = 123) was used to ensure reproducibility of results. To visualize the co-sensitization architecture across the identified clusters, a correlation network analysis was performed using the R package *qgraph*. A Pearson correlation matrix was computed between binary sensitization profiles for the 98 retained allergens across the 139 sensitized patients. A minimum correlation threshold of r ≥ 0.5 was applied for edge display, with edge thickness scaled proportionally to correlation strength (cut-off r = 0.6). Allergens with no connections at the r ≥ 0.5 threshold were excluded from the network.

To identify candidate confounders for the exploratory association between Cluster 1 and asthma prevalence, we compared Cluster 1 to the remaining cohort on age, sex, and urban/rural residence. Sex and urban/rural residence did not differ significantly between groups (Fisher’s exact test, p = 0.191 and p = 0.303, respectively), whereas age did (Wilcoxon test, p = 0.024). A *post-hoc* logistic regression model was therefore fitted with asthma as the outcome and Cluster 1 assignment (binary) and age (continuous) as predictors. Odds ratio (OR) with 95% confidence intervals (CI) were derived from the model coefficients.

### 2.4 Code availability

The complete R script required to reproduce the hierarchical clustering, dimensionality reduction, network graph generation, and statistical analyses is available in a public repository on GitHub: https://github.com/alexandrachera/ALEX-allergen-sensitization-clustering-analysis. The pipeline is designed to be executed using the anonymized dataset, which can be obtained from the corresponding author upon request.

## 3 Results

### 3.1 Cohort description

Of the 213 patients who underwent ALEX^2^ testing during the study period, 52 were excluded as they did not carry the diagnosis of AR, asthma and/or AD. After applying exclusion criteria, the remaining 161 atopic patients were included in the study population, of whom 139 had at least one detectable sensitization on ALEX^2^ testing and formed the final analytical cohort subjected to cluster analysis. The remaining 22 patients (13.7%) who showed no detectable sensitization on ALEX^2^ were only included in the descriptive statistical analysis, and were excluded from the cluster analysis.

The median age of the cohort was 35 years (IQR 26-48), with female predominance (64%, n = 103) and a predominantly urban residential background (85.9%, n = 134). Median tIgE was 203 kU/L (IQR 85.5, 1097).

AR was the most prevalent atopic disease, present in 135 patients (83.9%), followed by asthma in 64 patients (39.8%) and AD in 40 patients (24.8%). A substantial proportion of patients carried overlapping diagnoses (n = 67, 41.6%), including 36 patients with both AR and asthma, 18 with both AR and AD, 2 with both asthma and AD, and 11 patients carrying all three diagnoses simultaneously, consistent with the known co-occurrence of atopic diseases. Oral allergy syndrome was recorded as a clinical diagnoses in 11 patients (6.8%), prior to the multiplex assay. Among sensitized patients, the median number of positive ALEX^2^ results (extracts and molecular components combined) was 10 (IQR 4-15.5). Full patient characteristics are summarized in **Table 1**.

Within the analytical cohort of 139 patients, the youngest age group (18-24 years, n = 32) showed the highest median sensitization count (13, IQR 7-21.8), followed by the 25–34 year-old group (n = 36, with a median sensitization count of 10), whereas older groups showed median counts of 7, 9, and 8 for the 35-44, 45-54, and 55+ groups, respectively (**Figure 1B**). When comparing these discrete age groups, differences in sensitization breadth did not reach statistical significance (Kruskal-Wallis p=0.067). However, when analyzing age as a continuous variable across the analytical cohort, a statistically significant negative correlation was observed between age and sensitization breadth across the cohort (Spearman r = −0.265, p = 0.0016). TIgE values were evaluated in n = 137 patients with available measurements and did not follow a linear pattern across age groups: the 18–24 age group showed the highest median tIgE (671 kU/L), while the 55+ group also showed a comparatively elevated median tIgE (588 kU/L, IQR 105–2500 kU/L) despite reduced sensitization breadth, while the 35–44 and 45–54 groups showed the lowest median IgE values (138 and 187 kU/L, respectively).

**Figure 1:**
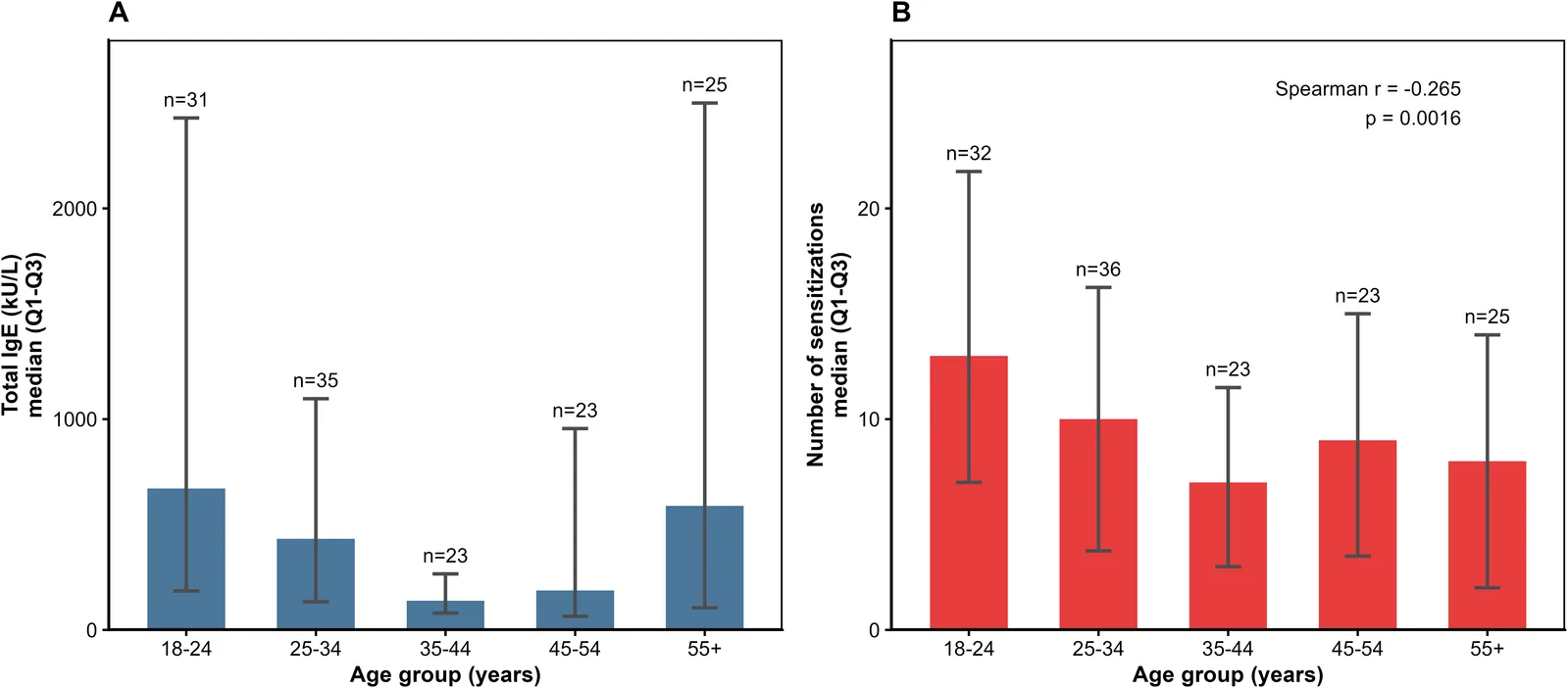
The relationship between age, total IgE, and number of sensitizations. **(A)** Shows the median tIgE values (kU/L) across age groups (n = 137: two patients lacked tIgE measurements). **(B)** Shows the number of allergen sensitizations across age groups (n = 139).

### 3.2 Molecular sensitization clusters

Silhouette analysis identified k = 4 as the solution with the highest average silhouette width (0.18, **Supplementary Figure S1**), yielding four clusters of sizes n = 89, 24, 16 and 10. However, the largest cluster of the k = 4 solution, comprising 89 of 139 patients (64%), resolved into two biologically distinct subgroups upon further examination. Specifically, at k = 5, this group was resolved into a minimally sensitized phenotype (n = 42) and a moderately polysensitized mixed inhalant phenotype (n = 47), producing five clusters of more balanced sizes (n = 42, 47, 24, 16, 10). The remaining three clusters from the k=4 solution (n = 24, 16, 10), corresponding to the grass pollen-dominant, ragweed/profilin-dominant and multi-panallergen phenotypes described below, were unaffected by this split.

Bootstrap stability analysis (1,000 resamples, Jaccard similarity) indicated that stability values differed across all clusters between the two solutions. Under k = 4, Jaccard means were 0.844 for the dominant cluster (n = 89), 0.513 for the grass pollen-dominant cluster (n = 24), 0.899 for the ragweed/profilin-dominant cluster (n = 16), and 0.764 for the multi-panallergen cluster (n = 10). Under k = 5, the grass pollen-dominant cluster (n = 24) showed a further decrease in stability (Jaccard mean = 0.392), while the ragweed/profilin-dominant cluster (n = 16) and the multi-panallergen cluster (n = 10) showed slight increases (Jaccard means 0.910 and 0.820, respectively). The two clusters formed by splitting the dominant k = 4 cluster (n = 89) showed reduced stability relative to their unsplit parent: the pauci-sensitized cluster (n = 42, Jaccard mean = 0.671), and the polysensitized cluster (n = 47, Jaccard mean = 0.564). Given the comparatively lower stability, clinical associations involving the grass pollen-dominant, the pauci-sensitized and polysensitized clusters, should be interpreted with appropriate caution. Notwithstanding this reduced stability, upon subsequent clinical characterization of the five resulting clusters, the minimally sensitized phenotype (n = 42) showed a significant one-vs-rest association with asthma that was not detectable at k = 4 (p = 0.023). This association is discussed further below and treated as exploratory given the non-significant global comparison and lack of FDR-corrected pairwise significance. The clinical and biological characteristics of each of the five clusters are illustrated in **Table 2**.

**Table 2:** Demographic, clinical, and sensitization characteristics of the exploratory five-cluster (k = 5) solution across the analysis cohort (n = 139), obtained after excluding the patients with no sensitizations identified through ALEX^2^ multiplex testing.

Characteristic	C1 (n=42)	C2 (n=24)	C3 (n=47)	C4 (n=10)	C5 (n=16)	p-value
Demographics						
Age, median (IQR)	42 (30, 55.8)	31 (25.8, 48)	31 (24, 44.5)	31 (24.8, 51.5)	43.5 (28.8, 51.5)	0.0926
Female, n (%)	29 (69%)	10 (41.7%)	29 (61.7%)	4 (40%)	12 (75%)	0.0955
Urban, n (%)	33 (80.5%)	19 (79.2%)	41 (91.1%)	10 (100%)	13 (81.2%)	0.327
**Total IgE kU/L, median (IQR)**	133.5 (74.5, 630.8)	681.5 (182.8, 1150.5)	634 (137.5, 2355)	196.5 (145.2, 502.8)	703.5 (207.5, 1589.8)	0.1172
**Median number of sensitizations (IQR)**	2 (1, 4)	13.5 (10.8, 16.2)	9 (5, 13.5)	14.5 (12.5, 26)	17 (14.8, 23.5)	**< 0.001**
Atopic diseases						
Allergic Rhinitis, n (%)	26 (61.9%)	24 (100%)	46 (97.9%)	10 (100%)	16 (100%)	**< 0.001**
Asthma, n (%)	23 (54.8%)	10 (41.7%)	15 (31.9%)	2 (20%)	5 (31.2%)	0.1335
Atopic Dermatitis, n (%)	13 (31%)	4 (16.7%)	7 (14.9%)	4 (40%)	5 (31.2%)	0.1864
Oral allergy syndrome, n (%)	1 (2.4%)	1 (4.2%)	3 (6.4%)	3 (30%)	3 (18.8%)	0.0255
OAS through PR-10, n (%)	0 (0%)	0 (0%)	0 (0%)	3 (30%)	0 (0%)	**< 0.001**
OAS through profilins, n (%)	0 (0%)	0 (0%)	0 (0%)	0 (0%)	3 (18.8%)	**0.0013**
OAS through nsLTP, n (%)	0 (0%)	0 (0%)	1 (2.1%)	2 (20%)	0 (0%)	0.0205

Median (Q1, Q3); n (%). Cluster structure reflects the exploratory k=5 solution (see Section 3.2); at the more stable k=4 solution, Clusters 1 and 3 form a single, undivided group (n=89). Continuous variables compared using Kruskal-Wallis test. Categorical variables compared using Fisher’s exact test with Monte Carlo simulation (B = 10,000). Bold p-values indicate statistical significance (p < 0.05). Patients may appear in more than one disease row due to overlapping diagnoses. OAS, oral allergy syndrome; nsLTP, non-specific lipid transfer protein; PR-10, pathogenesis-related protein 10; IgE, immunoglobulin E; IQR, interquartile range.

The sensitization profiles within the five clusters are visualized in **Figure 2**, which demonstrates distinct allergen sensitization patterns across the cohort. The grass pollen-dominant (n = 24) and ragweed/profilin-dominant (n = 16) clusters displayed the most highly concentrated sensitization patterns, whereas the paucisensitized group (n = 42) was characterized by near-universal absence of sensitization across all allergen families.

**Figure 2:**
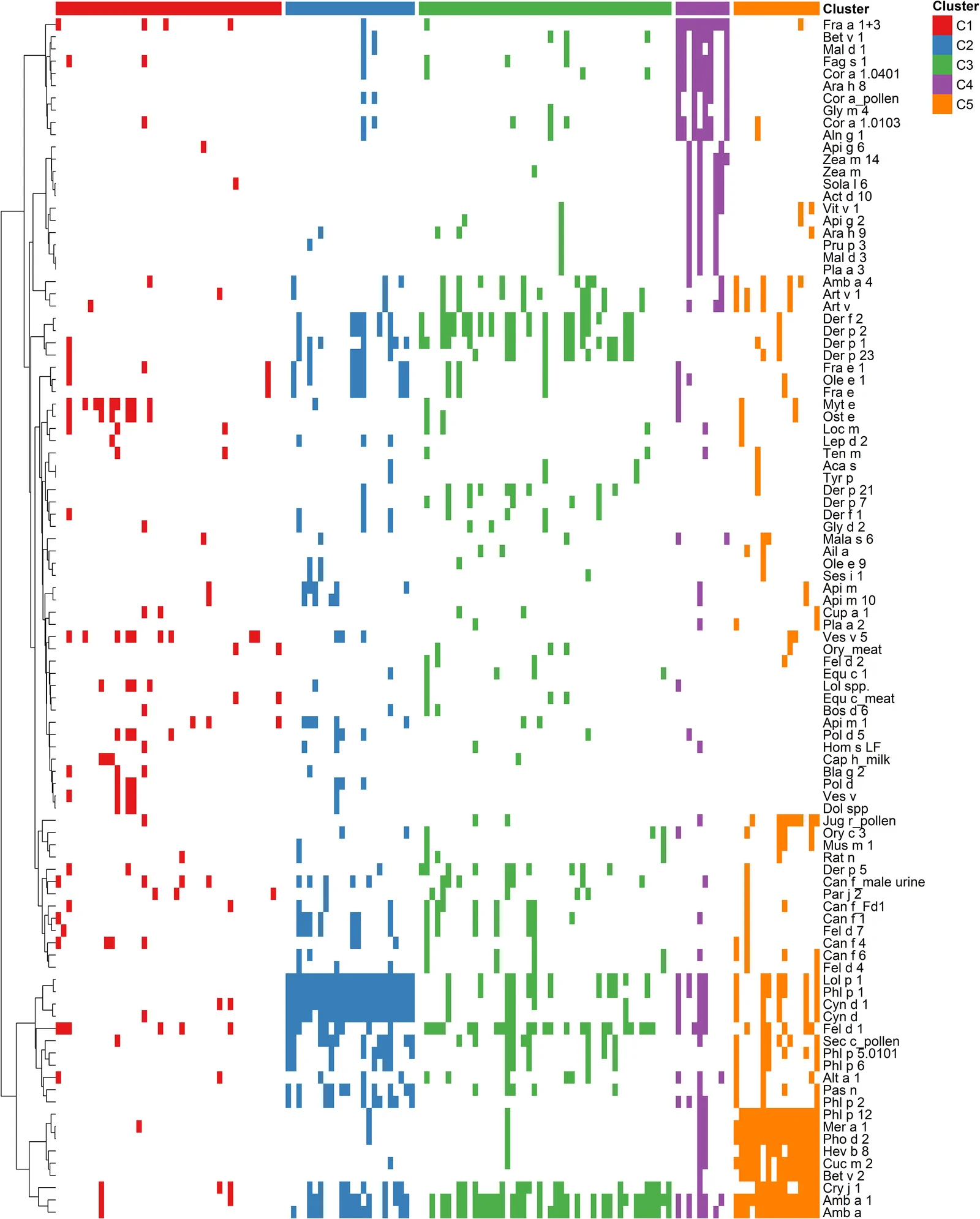
Heatmap of allergen sensitization profiles across the five exploratory clusters. Each column represents one patient (n = 139), and each row represents one of the 98 allergens retained for analysis. A colored cell indicates sensitization (sIgE ≥ 0.3 kU_A_/L), white indicates absence of sensitization. Cell color corresponds to cluster membership as indicated in the annotation sidebar. Patients are grouped by cluster assignment and separated by horizontal gaps; column ordering reflects Ward.D2 hierarchical clustering of allergens. Row dendrogram is not shown as patients are ordered by cluster membership for visual clarity.

UMAP projection of the binary sensitization matrix illustrated the spatial separation of the five clusters in two-dimensional space (**Figure 3**), providing a complementary, non-inferential visualization of the sensitization landscape. Consistent with its low sensitization count, the pauci-sensitized cluster (n = 42) occupied a distinct, isolated region separate from all other clusters. The grass pollen-dominant (n = 24) and polysensitized (n = 47) groups showed partial spatial overlap, reflecting their shared inhalant sensitizations, while the multi pan-allergen group (n = 10) remained spatially isolated, consistent with its unique PR-10 and nsLTP cross-reactivity profile. The ragweed/profilin-dominant group (n = 16) partially overlapped with the polysensitized phenotype (n = 47), indicating their shared sensitization patterns.

**Figure 3:**
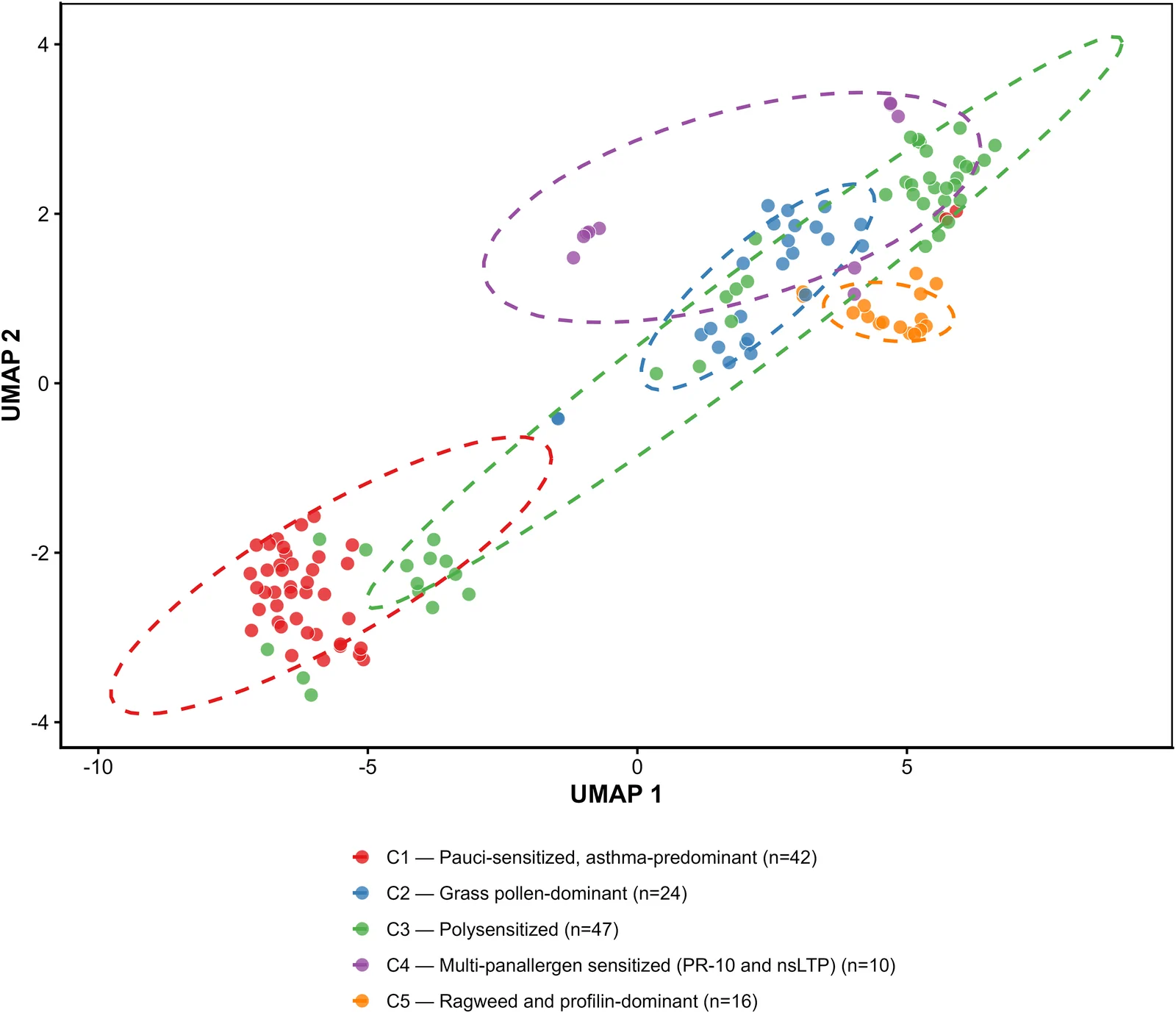
UMAP projection of binary allergen sensitization profiles colored by cluster assignment. Each point represents one patient (n = 139). UMAP was applied to the binary sensitization matrix (139 patients × 98 allergens) using Euclidean distance. Dashed ellipses represent descriptive concentration ellipses (68% of points under a bivariate normal assumption) for each cluster, and do not represent formal confidence regions for a population parameter. Color coding is consistent with **Figure 2**.

The co-sensitization network (**Figure 4**) illustrated the molecular architecture underlying the five clusters. In the full network (Panel A), five distinct communities of co-sensitizing allergens were visible, each corresponding to one of the identified clusters. The multi pan-allergen, namely PR-10 and nsLTP sensitized cluster (n = 10, C4, purple) and the ragweed and profilin-dominant cluster (n = 16, C5, orange) formed the most tightly interconnected communities, reflecting the strong cross-reactivity within these protein families. The grass pollen-dominant cluster (n = 24, C2, blue) showed a distinct community centered on major allergens such as Lol p 1, Phl p 1, Cyn d 1. The polysensitized group (n = 47, C3, green) exhibited broader connectivity across multiple allergen families, consistent with its mixed inhalant profile. The pauci-sensitized cluster (n = 42, C1, red) showed sparse connectivity, indicative of its low sensitization breadth. In the statistically filtered network (Panel B), the most robust co-sensitization patterns were concentrated within the PR-10/nsLTP, profilin/ragweed, and grass pollen communities, while a substantial proportion of allergens in the broader network did not reach cluster-specific statistical significance, reflecting the heterogeneous nature of the polysensitized (n = 47) and pauci-sensitized (n = 42) phenotypes.

**Figure 4:**
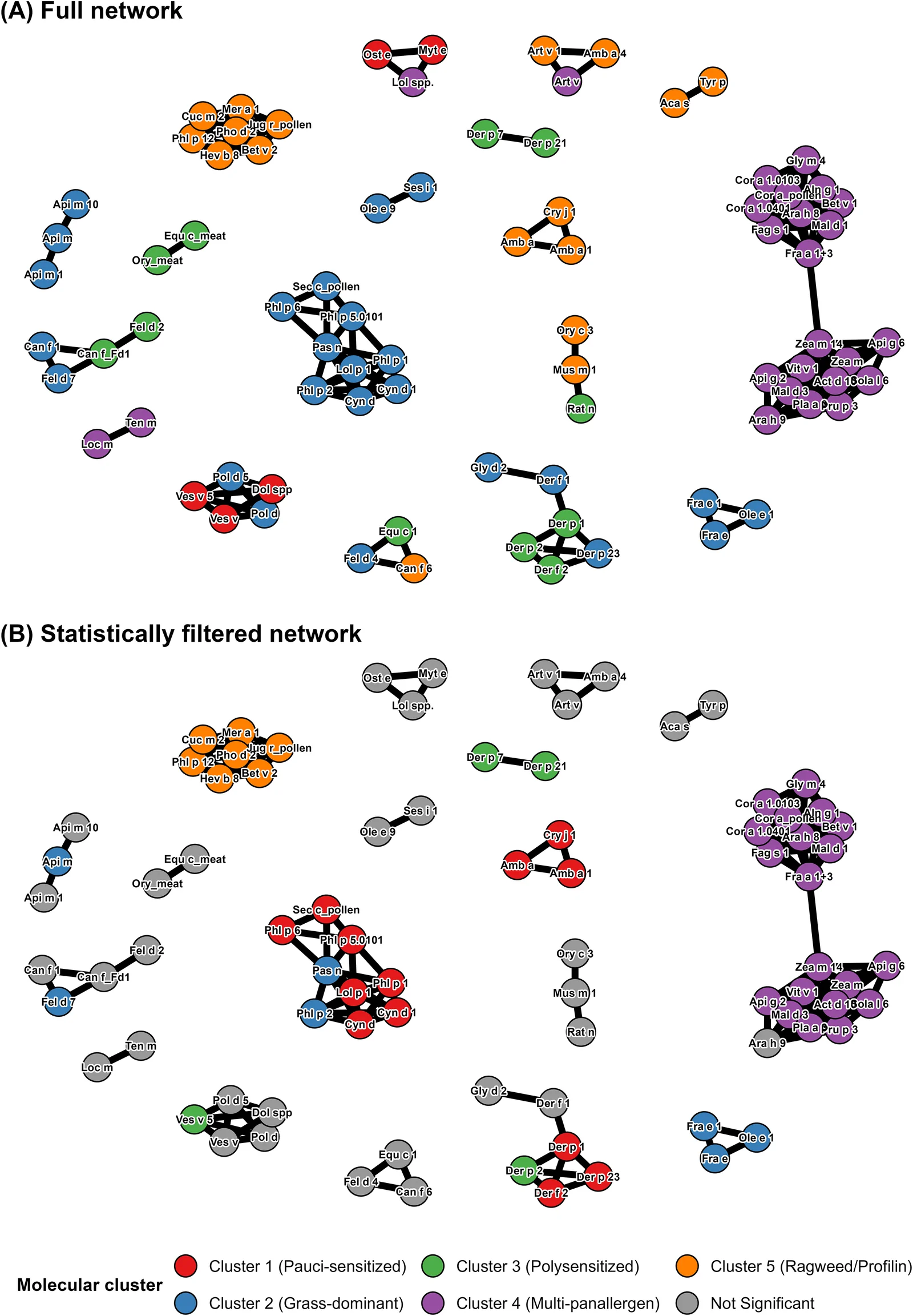
Cluster-based sensitization network of allergens. Each node represents one allergen and each edge represents a Pearson correlation between binary sensitization profiles across the 139 sensitized patients. Edges are displayed only when the correlation reaches a minimum inclusion threshold of r ≥ 0.5, and edge thickness is scaled proportionally to correlation strength up to r = 0.6, above which edges are displayed at maximum thickness. Allergens with no connections reaching the threshold are not displayed. **(A)** Full network: all nodes are color-coded according to their dominant cluster assignment (the cluster in which the allergen shows the highest sensitization prevalence), regardless of statistical significance. **(B)** Statistically filtered network: nodes are color-coded only where the allergen demonstrated a statistically significant association with the dominant cluster (one-vs-rest Fisher’s exact test, FDR-adjusted p < 0.05); allergens that did not reach statistical significance in any cluster are displayed in grey. Color coding is consistent across both panels and with **Figures 2** and **3**: red = Cluster 1 (Pauci-sensitized, asthma-predominant); blue = Cluster 2 (Grass pollen-dominant); green = Cluster 3 (Polysensitized); purple = Cluster 4 (Multi-panallergen sensitized - PR-10 and nsLTP); orange = Cluster 5 (Ragweed and profilin-dominant).

### 3.3 Clinical, biological and sensitization profiles across clusters

Five distinct clusters (n = 42, 47, 24, 16, 10) have been identified after performing the unsupervised hierarchical cluster analysis. While k = 4 represents the more stable, primary clustering solution (Section 3.2), the k = 5 solution is characterized in detail in our manuscript, since it resolves the heterogeneity which is clinically relevant within the dominant k = 4 cluster. Thus, findings specific to this five-way split should, therefore, be interpreted as exploratory rather than confirmatory.

Cluster 1, Pauci-sensitized, asthma-predominant (n = 42), was characterized by the lowest median number of sensitizations (2, IQR 1-4) and the lowest median tIgE (133.5 kU/L), yet carried the highest proportion of asthma patients (54.8%) and the lowest proportion of AR patients (61.9%) among all clusters, although the global comparison of asthma prevalence across clusters was not statistically significant (p = 0.1335, **Table 2**). Pairwise comparisons between clusters revealed that Cluster 1 had significantly lower AR prevalence compared to Clusters 2, 3, and 5 after FDR correction (**Supplementary Table S2**). Of note, high rates of asthma were also observed across our non-sensitized patients (40.9%, 9/22), with no statistically significant difference in asthma prevalence compared to Cluster 1 (Fisher’s exact test p = 0.430). Within cluster 1, a significant depletion of sensitization occurred across most allergen families, including grass pollens (Lol p 1, Phl p 1, Cyn d 1: 0-4.8%), HDM allergens (Der f 2, Der p 2: 0%) and cat dander (Fel d 1: 14.3%), consistent with its pauci-sensitized phenotype. All sensitization prevalences and corresponding FDR-adjusted p-values for each allergen showing at least one statistically significant cluster association (53 allergens in total) are presented in **Supplementary Table S3**.

Cluster 2, Grass pollen-dominant (n = 24), showed 100% AR prevalence with a moderate sensitization breadth (median 13.5) and was predominantly composed of younger patients (median age 31 years) (**Table 2**). Cluster 2 was defined by near-universal sensitization to grass pollen allergens (Lol p 1, Phl p 1, Cyn d 1: 100%; Cyn d: 95.8%), with additional sensitization to Pas n (45.8%), Sec c pollen (50%), and Fra e 1 (29.2%), consistent with a grass pollen-dominant profile (**Supplementary Table S3**).

Cluster 3, Polysensitized to both perennial and seasonal allergens (n = 47), similarly demonstrated near-universal AR prevalence (97.9%) with a broader mixed sensitization profile (median 9 sensitizations) and elevated tIgE values (median 634 kU/L) (**Table 2**). Cluster 3 showed significant enrichment for HDM allergens (Der f 2: 40.4%, Der p 1: 34%, Der p 2: 38.3%) alongside ragweed (Amb a 1: 66%) and cat dander (Fel d 1: 51.1%), reflecting a mixed perennial and seasonal polysensitized phenotype (**Supplementary Table S3**).

Cluster 4, Multi-panallergen sensitized (PR-10 and nsLTP) (n = 10), was the smallest cluster and was notable for the highest rates of OAS (30%, p = 0.026) among all clusters (**Table 2****).** The molecular profile of Cluster 4 was defined by two co-occurring panallergen families: PR-10 allergens, with 100% sensitization to Fra a 1 + 3 and 70% sensitization to Bet v 1, Cor a 1.0401, Fag s 1, Aln g 1, and Ara h 8; and nsLTP allergens, including Vit v 1, Api g 2, Pru p 3, Mal d 3, Pla a 3, Zea m 14, Act d 10, Api g 6, and Sola l 6 (**Supplementary Table S3**). The OAS subtype distribution in Cluster 4, derived by integrating clinical OAS diagnoses with molecular endotyping from ALEX^2^, was consistent with its molecular profile, with significant enrichment for PR-10-associated OAS (30%, p < 0.001) and nsLTP-associated OAS (20%, p = 0.021), and with no profilin-associated OAS detected in any patient of this cluster.

Cluster 5, Ragweed and profilin-dominant (n = 16) showed the highest sensitization breadth (median 17, IQR 14.8–23.5) and the highest median tIgE (703.5 kU/L, IQR 207.5–1589.8) among all clusters, with universal AR prevalence (100%) and high rates of OAS (18.8%) (**Table 2**). The molecular profile was characterized by universal sensitization to ragweed (Amb a 1: 100%; Amb a extract: 93.8%) and six profilin markers: Mer a 1 and Pho d 2 (100%), Phl p 12 (93.8%), Cuc m 2 (87.5%), and Hev b 8 and Bet v 2 (81.2%) (**Supplementary Table S3**). The clinical OAS diagnoses enriched by the subtype distribution clarified by the multiplex assay were exclusively profilin-associated in this cluster (18.8%, p = 0.001), with no PR-10 or nsLTP-associated OAS detected in any patient of this cluster.

Cluster assignment varied across clinical comorbidity profiles. Patients without AR were almost entirely grouped in Cluster 1: 6/6 (100%) with isolated AD, 2/2 (100%) with asthma and AD, and 8/9 (88.9%) with isolated asthma. Among the 62 patients presenting with overlapping diagnoses (44.6% of the analytical cohort), those with both AR and asthma were concentrated in Clusters 1, 2, and 3 (31.4%, 22.9%, and 31.4%), with only one patient assigned to Cluster 4 (2.9%), whereas patients with AR and AD showed a broader spread and were relatively overrepresented in Clusters 4 and 5 (18.8% and 25.0%). This finding did not reach statistical significance (Fisher’s exact test, p = 0.21), so it should be regarded as exploratory. Patients carrying the full atopic triad (n = 9 in the analytical cohort of 139 patients) were distributed across all five clusters without concentration, but showed the highest sensitization breadth of any comorbidity group (median 14, IQR 13–29).

A statistically significant positive correlation was observed between the number of allergen sensitizations and tIgE levels across the analytical cohort (Spearman r = 0.463, p < 0.001), supporting the idea that sensitization breadth is a meaningful biological correlate of tIgE production. This relationship varied across clusters (Cluster 1: r = 0.260, p = 0.096; Cluster 2: r = 0.311, p = 0.159; Cluster 3: r = 0.675, p < 0.001; Cluster 4: r = 0.744, p = 0.014; Cluster 5: r = 0.533, p = 0.034), with significant associations in Clusters 3, 4 and 5 and weaker, non-significant relationships in Clusters 1 and 2. Cluster 4 (Multi-panallergen sensitized) showed the steepest association despite only moderate absolute tIgE, though with n = 10 this estimate is imprecise; its extensive apparent sensitization breadth likely reflects panallergen cross-reactivity rather than primary nature of panallergen sensitizations.

## 4 Discussion

Using hypothesis-free unsupervised hierarchical clustering of ALEX^2^ multiplex sensitization data, we identified four primary molecular sensitization clusters in an adult atopic cohort consisting of AR, asthma and/or AD patients diagnosed by an Allergy specialist, with an exploratory five-cluster refinement further resolving clinically relevant heterogeneity within the dominant cluster. Understanding sensitization patterns may aid in informing strategies for more targeted testing and AIT selection, while also providing baseline data to be monitored over time in order to observe whilethe impact of environmental change on allergic sensitization (12). To our knowledge, this represents the first such analysis using the ALEX^2^ platform in a Romanian atopic population. The present cross-sectional analysis did not evaluate AIT selection, response, or longitudinal therapeutic outcomes.

The five clusters broadly align with sensitization phenotypes reported across European and global cohorts, typically comprising a low-sensitization group, a predominant pollen group, and a large multiple sensitization group (‘broad’) of varying severity (1, 2, 7, 14), while also revealing phenotypes specific to the Romanian and Eastern European landscape of allergen exposure. The absence of a distinct HDM-dominant cluster in our study is explicable since HDM sensitization was highly prevalent across all five clusters in our cohort rather than being concentrated in a specific subgroup, suggesting that in our urban Romanian population HDM functions as a background sensitization present across multiple phenotypes rather than as a defining cluster characteristic. This pattern differs from Northern and Western European cohorts where HDM-dominant clusters are consistently identified, and may reflect differences in exposure patterns or the competing dominance of ragweed as the primary seasonal sensitizer in this geographic context.

Sex distribution did not differ significantly across the five clusters (p = 0.096), despite a female predominance in the overall cohort (64%), and in contrast to studies reporting male predominance for most allergen families (27). A trend toward male predominance was observed in Cluster 2 (grass pollen-dominant, 58.3% male, p = 0.065), consistent with reported higher grass pollen sensitization rates in males, but did not reach statistical significance, likely reflecting limited statistical power in this cluster. These findings suggest that molecular sensitization phenotypes in adult atopic patients may be driven primarily by environmental exposure and immune mechanisms rather than sex-related factors.

A significant negative correlation between age and number of sensitizations was observed (Spearman r = -0.265, p = 0.0016), and it is consistent with population-based data from Czolk et al. (24), who identified the highest sensitization burden in the youngest adult age group (25–34 years) and attributed this to the fact that younger generations grew up during a period of rapid urbanization, increased allergen exposure, and lifestyle changes associated with rising atopy prevalence, while older generations were less exposed to these environmental shifts during their immunologically formative years. This age-related attenuation of sensitization breadth likely also reflects progressive immunological maturation and the known tendency for IgE-mediated sensitization to narrow with advancing age even as atopic disease persists. However, in the same year, Zhang et al. (29) reported that polysensitization probability increased with rising age and cumulative allergen exposure in a Chinese asthma cohort, suggesting that the relationship between age and sensitization breadth may differ across geographic regions, allergen environments, and study populations, and warrants further investigation. Notably, our group of older patients (55+ years old) showed a trend toward elevated median tIgE despite comparatively few allergen sensitizations, although differences in sensitization breadth across age groups did not reach statistical significance (Kruskal-Wallis p = 0.067). These patients were distributed across all five clusters, with nearly half assigned to Cluster 1 (12/25, 48%; C2 n = 2, C3 n = 4, C4 n = 3, C5 n = 4). The elevation was most marked within Cluster 1, where patients aged 55 years and over had a median tIgE of 1490 kU/L against a median of 2 sensitizations, compared with a cluster-wide median of 133.5 kU/L. Given the small numbers within each age–cluster stratum, these values are descriptive only. Nonetheless, the pattern does not appear attributable to over-representation of the polysensitized cluster, and may suggest elevated total IgE in the absence of broad specific sensitization among older patients in this cohort. Age-related alterations in immune function, including immunosenescence, introduce complex changes in IgE regulation that are not fully understood, and the relationship between tIgE and allergen-specific sensitization may differ substantially in older compared to younger atopic patients (39). The mechanisms underlying this dissociation therefore remain incompletely understood and warrant dedicated investigation. This observation nonetheless highlights the limitation of tIgE as a standalone diagnostic marker in older patients and supports the value of molecular multiplex testing for a more accurate characterization of the individual sensitization landscape.

The high prevalence of asthma (54.8%) in our pauci-sensitized Cluster 1 (n = 42) might appear paradoxical in the context of studies reporting that greater sensitization breadth was associated with higher asthma risk. Beck et al. (5) found that 91% of children hospitalized for asthma were sensitized to at least one allergen and 68% to three or more, while Howard et al. (22) demonstrated that within-cluster sensitization breadth in early childhood correlated with asthma prevalence at ages 5 and 16. Both studies, however, focused on pediatric populations selected for monitoring disease severity or followed longitudinally from birth, with a high probability of enriched IgE-mediated allergic mechanisms. Our cohort consists of an adult, tertiary-center cohortcenter may capture a broader phenotypic spectrum including non-allergic asthma phenotypes underrepresented in pediatric severity-based cohorts. However, our finding should be regarded as exploratory, since the overall comparison across all five clusters was not significant (p = 0.1335, **Table 2**), and no pairwise comparison with Cluster 1 survived FDR correction (**Supplementary Table S2**). Asthma was also present in 40.9% of non-sensitized patients excluded from clustering analysis, a rate not statistically different from Cluster 1, suggesting this enrichment may not be specific to this cluster alone. As Cluster 1 patients were also older than the rest of the cohort (p = 0.024), we tested for confounding by age, with the association persisting after adjustments (OR = 2.18, 95% CI 1.02-4.66, p = 0.045, not adjusted for multiple comparisons), though as a *post-hoc* test in a modestly sized subgroup, this should not be taken as definitive. To further assess whether this association reflected a pattern already present prior to the k=4 -to-k=5 split, we examined asthma prevalence in the undivided dominant cluster identified at k = 4 (n=89). Asthma prevalence in this unsplit group was 42.7%, compared to 34.0% in the remaining k = 4 clusters (Fisher’s exact test, OR = 1.44, 95% CI [0.67, 3.19], p = 0.368, not adjusted for multiple comparisons). Because this difference was not statistically significant, this descriptive comparison should be interpreted as strictly exploratory context for the subsequent Cluster 1 finding. Evidence from multiple European cohorts further suggests that mechanisms responsible for asthma development differ from those driving disease severity, and that sensitization patterns associated with asthma diagnosis are not necessarily informative about severity (6, 8, 19). The important feature of IgE response linked to increased asthma risk is not individual sIgE to any single allergen but the pattern of interactions between different sIgE, with some connectivities increasing and others decreasing asthma risk (7). A low-sensitization group associated with absence of asthma and AR has been described in the population-based EGEA cohort (18), contrasting with our exploratory finding, a discrepancy likely reflecting differences between population-based and referred clinical cohorts. Due to the fact that allergic IgE-mediated asthma represents only one subset within the broader Type 2 (T2) inflammatory spectrum, and non-T2 pathways, such as Th1 or Th17, can also drive disease, our data do not allow for a definitive endotypic classification of these pauci-sensitized patients (40). Distinguishing non-IgE T2 mechanisms, such as IL-5-driven eosinophilic inflammation, from non-T2 pathways in this group will require longitudinal studies alongside complementary biomarkers, such as Fractional Exhaled Nitric Oxide (FeNO) and blood eosinophil counts.

Cluster 2 (Grass pollen-dominant, n = 24) was defined by near-universal grass pollen sensitization and predominantly affected younger adults with universal AR prevalence. Grass pollen sensitization is among the most prevalent sensitization patterns globally, described consistently across European and non-European cohorts (3, 12, 20). The temporal dimension of grass sensitization appears particularly important: early-onset trajectories starting in preschool age have been associated with subsequent asthma, while late-onset trajectories are more strongly associated with AR (8, 20). Importantly, Howard et al. (22) demonstrated that 5-year-old patients from the grass pollen and cat allergen cluster were linked to developing asthma at age 16, but only when Fel d 1 co-sensitization was present, as isolated grass pollen sensitization was not sufficient to drive the asthma association. The relatively low cat allergen sensitization in our Cluster 2 is therefore consistent with an AR-predominant rather than asthma-predominant phenotype, supporting a late-onset or persistent AR trajectory rather than an early-onset asthma-associated profile. Cross-reactivity among grass pollen allergens is extensive and well documented across diverse geographic settings (12), consistent with the broad grass pollen sensitization profile observed in our patients.

Cluster 3 (n = 47), characterized by broad sensitization to both perennial and seasonal allergens, represents the polysensitized phenotype consistently described across published clustering studies, universally associated with higher disease burden, elevated tIgE, and more frequent atopic comorbidities (2, 6, 7, 17, 19). Our Cluster 3 showed near-universal AR prevalence (97.9%) and the highest tIgE among the five clusters (median 634 kU/L), consistent with these findings. Atopic multimorbidity, however, was not correspondingly elevated: 18 of 47 patients (38.3%) carried two or more atopic diagnoses, the lowest proportion among the five clusters (range 38.3–56.2%), and the majority (28/47) presented with AR alone.

Cluster 4 (Multi-panallergen sensitized, n = 10) was characterized by co-sensitization to both PR-10 and nsLTP panallergen families, and was associated with the highest rates of pre-recorded, clinically established OAS (30%) among all five clusters. PR-10 proteins, expressed in high concentrations in pollen, seeds, and fruits, are frequently responsible for cross-sensitization and OAS through their homologs in plant-derived foods (9). The simultaneous presence of PR-10 and nsLTP sensitization is clinically relevant because these families mediate distinct OAS phenotypes with different severity profiles: PR-10-mediated OAS is typically mild and localized, while nsLTP-mediated OAS can be systemic and associated with anaphylaxis. The co-occurrence of both families, consistent with our OAS subtype analysis showing enrichment for PR-10-associated (30%, p < 0.001) and nsLTP-associated OAS (20%, p = 0.021) with complete absence of profilin-associated OAS, is consistent with the ‘multi-panallergen pollen-food syndrome’ endotype described by Mastrorilli et al. (25) in the I-PAN (Italian Pediatric Allergy Network) pediatric network, associated with the most severe clinical presentation and multiple offending foods. The SAMP cohort identified a PR-10-dominant cluster in school-age Parisian children as well, its increasing prevalence being linked to global warming and longer pollination seasons (2, 41). Notably, despite broad apparent sensitization, tIgE was moderate in Cluster 4 (median 196.5 kU/L), explainable through the cross-reactive nature of PR-10 and nsLTP sensitizations, where a single primary sensitization to birch pollen generates multiple apparent positive results through molecular mimicry, without necessarily producing the high tIgE levels associated with true co-sensitization. The fact that this endotype is identifiable in our adult cohort suggests that the multi-panallergen sensitization pattern is a stable, biologically coherent phenotype that persists into adulthood and is detectable across different geographic and age contexts.

Cluster 5 (Ragweed and profilin-dominant, n = 16) represents the most geographically specific finding of our study. Defined by universal ragweed sensitization (Amb a 1: 100%) and profilin co-sensitization across six molecular markers (Mer a 1, Pho d 2, Phl p 12, Cuc m 2, Hev b 8, Bet v 2), this cluster is biologically consistent with the unique allergenic environment of the Pannonian plain, where Ambrosia artemisiifolia constitutes one of the highest pollen burdens in Europe (34). However, as individual-level pollen exposure and longitudinal aerobiological data were not included in the cluster analysis, this environmental interpretation remains inferential rather than directly demonstrated. Ragweed, native to North America, has become invasive across Europe with sensitization prevalence ranging from 19.5% in Southern Bavaria to 60% in Hungary (32), while in the Black Sea region has been identified as the most important seasonal allergen with the strongest statistical association with asthma development (35). In Romania, AR induced by sensitization to ragweed pollen has been most frequently reported in the North-West region (33). Geographic and climatic factors determine which allergenic plants are characteristic for a given area, explaining why hypersensitivity patterns differ between regions (10), and ongoing climate change is further driving ragweed habitat expansion across Europe (34, 35). The profilin-associated OAS observed exclusively in this cluster (18.8%, p = 0.001), with complete absence of PR-10 or nsLTP-associated OAS, corresponds precisely to the ‘profilin pollen-food syndrome’ endotype described by Mastrorilli et al. (25), characterized by OAS triggered by Cucurbitaceae, consistent with the high prevalence of melon allergen (Cuc m 2) sensitization of 87.5% in this cluster. Unlike Cluster 4, Cluster 5 patients present with a more restricted food allergy profile, reflecting the narrower cross-reactive network of profilins compared to PR-10 and nsLTP allergens. However, because OAS subtypes were defined by integrating the current multiplex molecular profiling with patient-reported histories, and because these findings are based on very small absolute numbers of affected patients (2–3 per significant cell in Clusters 4 and 5), these OAS subtype associations should be interpreted as exploratory rather than definitive, despite reaching statistical significance after FDR correction; replication in larger, independent cohorts is needed before these subtype-specific associations can be considered robust. The identification of this cluster in a Romanian adult cohort, where similar profilin-ragweed profiles have not been systematically described using molecular multiplex testing, represents a geographically relevant contribution to the understanding of atopic phenotypes in Central and Eastern Europe.

## 5 Strengths and limitations

The present study has several strengths. To our knowledge, it represents the first unsupervised learning analysis of molecular allergen sensitization profiles in a Romanian adult atopic cohort using the ALEX^2^ multiplex platform, providing region-specific data from a geographic area largely absent from the existing literature. The inclusion of the full atopic triad rather than a single disease allows for a more comprehensive characterization of sensitization phenotypes across the atopic spectrum. The use of hierarchical clustering without prior dimensionality reduction preserves the full variance of the sensitization data and facilitates clinical interpretability of the resulting clusters. Although formal external validation against raw data from independent cohorts was not performed, the identified clusters were systematically compared with findings from over 26 published sensitization profiling studies across Europe, Asia, North America, and Africa, providing broad contextual support for the identified phenotypes within the existing literature.

Several limitations must be acknowledged. As a cross-sectional study, the present analysis provides a single time-point snapshot of sensitization profiles and cannot establish causal relationships between sensitization patterns and atopic disease outcomes, nor track changes in cluster membership over time.

The absence of objective clinical severity metrics, including spirometry parameters, FeNO, exacerbation rates for asthma patients, and disease-specific severity scores, such as the Asthma Control Test (ACT), the Visual Analogue Scale (VAS) for assessing the clinical severity of AR, or the Eczema Area and Severity Index (EASI) and SCORing Atopic Dermatitis (SCORAD), limits the clinical anchoring of the identified phenotypes and does not allow for a clear assessment of differences in disease severity between clusters (2, 6). We are currently updating our database with these clinical variables, and a subsequent analysis on an expanded cohort with comprehensive clinical data is planned.

The final number of clusters (k = 5) was selected on the basis of biological interpretability rather than mathematical optimality; even though the k = 4 solution showed a higher average silhouette width (0.18 vs. 0.08), bootstrap stability analysis confirmed that this choice involves a trade-off, and a sensitivity analysis comparing the Euclidean-based clustering solution to an alternative based on Jaccard distance showed only modest overall agreement (ARI = 0.261). Cluster 1 (pauci-sensitized) and Cluster 2 (grass pollen-dominant) showed comparatively higher concordance between the two-distance metrics (74% and 87.5% of patients retained in the corresponding Jaccard cluster, respectively), while Clusters 3, 4, and 5 showed greater sensitivity to this analytical choice, with Cluster 3 in particular fragmenting across multiple Jaccard-derived groups. This indicates that shared absence of sensitization may contribute meaningfully to the Euclidean-based clustering structure, particularly outside of Cluster 1, and that the boundaries of the polysensitized and panallergen-dominant clusters should be interpreted with corresponding caution.

The relatively small sample size, particularly in Clusters 4 and 5 (n = 10 and n = 16 respectively), limits statistical power for pairwise comparisons and makes comparisons with other groups difficult (2).

The age-adjusted odds ratio reported for the association between Cluster 1 and asthma reflects a single, *post-hoc* test not corrected for multiple comparisons, and should be interpreted as hypothesis-generating rather than confirmatory.

Allergens with a prevalence below 3% in the analytical cohort were excluded prior to clustering, a necessary step to reduce noise and the impact of zero-inflated variables on cluster accuracy (6), though this filtering may have excluded some informative allergens.

Our cohort was evaluated between January 2024 and May 2026 using the ALEX^2^ macroarray, which was the only multiplex test available at our clinical center during this time period. While ALEX^2^ provided a 295-allergen molecular profile covering the dominant primary and cross-reactive sensitization patterns relevant for our cluster analysis, the subsequent ALEX^3^ platform includes an expanded set of molecular components and may therefore capture sensitizations not represented in our cohort. Sensitization breadth was calculated as the number of positive ALEX^2^ results including both allergen extracts and molecular components.

Both allergen extracts and molecular components were included as separate variables in the clustering matrix, and while this ensures that sensitization is captured even when a patient reacts to an extract without testing positive for the specific corresponding component (or vice versa), it may give allergen sources represented by multiple correlated variables, such as ragweed, disproportionate weight in the distance-based clustering relative to sources represented by a single variable. To quantify this, we examined extract-component discordance for the major ragweed allergen: of 64 patients sensitized to ragweed extract (Amb a), 60 (93.8%) were also positive for the major component Amb a 1, with only 4 (6.3%) extract-positive but component-negative, indicating close concordance for this dominant, primary sensitizing component, as expected. In contrast, a minor ragweed component (Amb a 4) showed substantially greater discordance within the same extract-positive population (only 15 of 64 patients concordant).To directly test whether this disproportionate weighting affected the clustering structure, we constructed a reduced matrix collapsing the 8 allergen sources represented by both an extract and one or more components (Amb a, Api m, Art v, Cyn d, Fra e, Pol d, Ves v, Zea m) into a single binary variable per source (88 variables total, down from 98), and re-ran the identical Ward.D2/Euclidean clustering pipeline. Agreement with the original solution was moderate overall (adjusted Rand index = 0.488), but varied substantially by cluster: the pauci-sensitized cluster (n = 42), grass pollen-dominant cluster (n = 24), and ragweed/profilin-dominant cluster (n = 16) were highly concordant between the two solutions (100%, 95.8%, and 93.75% of patients preserved, respectively). Conversely, the polysensitized cluster (n = 47) showed a tendency to fragment, and the multi-panallergen cluster (n = 10) split roughly evenly between two groups. Combined with its comparatively lower bootstrap stability and greater sensitivity to the choice of distance metric reported above, the boundaries of the polysensitized cluster appear consistently less robust across multiple independent sensitivity checks, and clinical associations involving this group should be interpreted with corresponding caution.

The use of different unsupervised learning methods, variable sets, and encodings can lead to inconsistent subgroupings, and more careful and consistent selection of markers across populations is required before definitive conclusions can be drawn from unsupervised learning analyses in atopic disease (3, 42).

As ALEX^2^ testing was ordered at the recommendation of Allergy specialists for patients considered to require extended molecular evaluation, our findings reflect a selected tertiary-care cohort rather than a population-based sample, and may not generalize to the broader Romanian or Eastern European atopic population. Single-center recruitment further limits generalizability of our selected tertiary-care cohort findings to other Romanian or European populations, and direct external validation in an independent cohort is warranted as a priority for future work.

## 6 Conclusion

The present study identified four primary molecular allergen sensitization clusters in an adult atopic cohort from Romania, with an exploratory, five-cluster refinement further resolving heterogeneity within the dominant cluster, using hierarchical clustering of ALEX^2^ multiplex data. To our knowledge, this is the first unsupervised clustering analysis of molecular allergen sensitization profiles across the full atopic triad in an adult Romanian cohort. The identified phenotypes, namely a pauci-sensitized cluster with an exploratory enrichment for asthma, a grass pollen-dominant cluster, a ‘broad’, polysensitized cluster, a multi-panallergen cluster defined by PR-10 and nsLTP co-sensitization, and a ragweed pollen and profilin-dominant cluster, broadly align with sensitization profiles described in other European and global cohorts, while also reflecting the specific allergenic landscape of the Pannonian plain region. The ragweed/profilin-dominant phenotype and the multi-panallergen phenotype, supported by their mutually exclusive OAS subtype distributions and their correspondence with established OAS endotypes, represent the most geographically and clinically specific contributions of this work. The exploratory association between asthma and the pauci-sensitized cluster illustrates that the relationship between IgE sensitization and atopic disease is complex and cannot be reduced to overall sensitization counts, though this observation will require confirmation in future studies incorporating endotypic biomarkers. Component-resolved multiplex testing may help guide the interpretation of cross-reactive sensitization patterns and could inform future approaches to AIT selection and endotype-based treatment stratification, pending prospective validation incorporating treatment outcome data. Such validation should be pursued in larger, independent cohorts, alongside incorporation of objective clinical severity metrics, and longitudinal follow-up to establish whether the identified sensitization phenotypes predict disease trajectory and therapeutic response, or whether these molecular profiles evolve over time. The ongoing expansion of our cohort and collection of clinical outcome data will allow for a more comprehensive assessment of the clinical relevance of these molecular sensitization phenotypes in adult atopic disease.

## Data Availability

The raw data supporting the conclusions of this article will be made available by the authors, without undue reservation.
