# Supplementary material for: Molecular sensitization clusters in adult atopy: an ALEX2-based analysis from Eastern Europe
Source: Front Immunol. 2026 Sep 18;17:1960324. doi: 10.3389/fimmu.2026.1960324 (PMC13630640; doi:10.3389/fimmu.2026.1960324)
Supplement: Supplementary file 1 [file DataSheet1.docx]

**SUPPLEMENTARY MATERIALS**

**Molecular Allergen Sensitization Clusters in Adult Atopy: An ALEX^2^-Based Analysis from Eastern Europe**

*Alexandra Chera*^1,2,3^*, Selda Ali*^1,2,*^*, Courtney Chau*^4^*, Benjamin Hu*^4^*, Alanis Villanueva*^4^*, Emma Guttman-Yassky*^4^*, Octavian Bucur*^3^*, Roxana-Silvia Bumbacea*^1,2^

^1^Carol Davila University of Medicine and Pharmacy, Bucharest, Romania

^2^Department of Allergy and Clinical Immunology, ‘Dr. Carol Davila’ Clinical Nephrology Hospital, Bucharest, Romania

^3^Genomics Research and Development Institute, Bucharest, Romania

^4^Department of Dermatology, Icahn School of Medicine at Mount Sinai, New York, NY USA

*** Corresponding author**: Lecturer Selda Ali, MD, Carol Davila University of Medicine and Pharmacy, Bucharest, Romania; Department of Allergy and Clinical Immunology, ‘Dr. Carol Davila’ Clinical Nephrology Hospital, Bucharest, Romania. **E-mail:** selda.ali@umfcd.ro.

***Short title:*** *Adult Atopy Molecular Sensitization Clusters*

**Figure S1. Average silhouette width for hierarchical clustering solutions with k = 2 to 8 clusters.** Average silhouette width was calculated for each clustering solution (k = 2 to 8) derived from Ward.D2 hierarchical clustering applied to the binary sensitization matrix of 139 atopic patients across 98 allergens. The silhouette width quantifies the degree of separation between clusters, with higher values indicating greater within-cluster cohesion and between-cluster separation. The red dashed line indicates the primary solution at k = 4, which yielded the highest average silhouette width (0.18). An exploratory five-cluster refinement (k = 5, silhouette width = 0.08) was characterized in detail throughout the article, as it resolved clinically relevant heterogeneity not captured by the k = 4 solution, as detailed in the as detailed in the Results section. The overall low silhouette values are consistent with previously reported findings in binary allergen sensitization datasets and reflect the inherent geometric overlap of sensitization profiles rather than the absence of biologically meaningful cluster structure.

**Figure S2. Relationship between allergen sensitization breadth and total IgE levels across the five exploratory clusters.** Each point represents one sensitized patient (n = 137 2 patients were excluded due to missing total IgE values). The x-axis shows the total number of allergen sensitizations(sIgE ≥ 0.3 kU_A_/L) and the y-axis shows total IgE on a logarithmic scale. Linear regression lines with 95% confidence intervals are shown for each cluster for visualisation only; the correlation statistics reported in the main text are Spearman rank coefficients (full cohort r = 0.463, p < 0.001; per-cluster values in Section 3.3). Regression estimates for Clusters 4 (n = 10) and 5 (n = 16) are based on small samples and should be interpreted with caution. Color coding is consistent with Figures 2-4.

# **Table S1. Allergens retained for cluster analysis (n = 98).**

| **Rank** | **Allergens** | **N sensitized** | **Prevalence (%)** |
| --- | --- | --- | --- |
| 1 | Amb a 1 | 66 | 47.5 |
| 2 | Amb a | 64 | 46.0 |
| 3 | Fel d 1 | 49 | 35.3 |
| 4 | Lol p 1 | 47 | 33.8 |
| 5 | Phl p 1 | 46 | 33.1 |
| 6 | Cyn d 1 | 39 | 28.1 |
| 7 | Cry j 1 | 37 | 26.6 |
| 8 | Cyn d | 36 | 25.9 |
| 9 | Sec c_pollen | 29 | 20.9 |
| 10 | Der f 2 | 26 | 18.7 |
| 11 | Der p 1 | 26 | 18.7 |
| 12 | Der p 2 | 25 | 18.0 |
| 13 | Mer a 1 | 21 | 15.1 |
| 14 | Pas n | 21 | 15.1 |
| 15 | Phl p 5.0101 | 21 | 15.1 |
| 16 | Der p 23 | 20 | 14.4 |
| 17 | Pho d 2 | 20 | 14.4 |
| 18 | Phl p 12 | 19 | 13.7 |
| 19 | Amb a 4 | 18 | 12.9 |
| 20 | Can f_male urine | 18 | 12.9 |
| 21 | Cuc m 2 | 18 | 12.9 |
| 22 | Alt a 1 | 17 | 12.2 |
| 23 | Art v 1 | 17 | 12.2 |
| 24 | Can f 1 | 17 | 12.2 |
| 25 | Fra a 1+3 | 17 | 12.2 |
| 26 | Phl p 2 | 17 | 12.2 |
| 27 | Art v | 16 | 11.5 |
| 28 | Hev b 8 | 16 | 11.5 |
| 29 | Fel d 7 | 15 | 10.8 |
| 30 | Ole e 1 | 15 | 10.8 |
| 31 | Phl p 6 | 15 | 10.8 |
| 32 | Bet v 2 | 14 | 10.1 |
| 33 | Can f_Fd1 | 14 | 10.1 |
| 34 | Der p 5 | 14 | 10.1 |
| 35 | Fra e 1 | 14 | 10.1 |
| 36 | Myt e | 14 | 10.1 |
| 37 | Ves v 5 | 14 | 10.1 |
| 38 | Cor a 1.0103 | 13 | 9.4 |
| 39 | Fag s 1 | 13 | 9.4 |
| 40 | Bet v 1 | 12 | 8.6 |
| 41 | Fra e | 12 | 8.6 |
| 42 | Jug r_pollen | 12 | 8.6 |
| 43 | Can f 4 | 11 | 7.9 |
| 44 | Can f 6 | 11 | 7.9 |
| 45 | Cor a 1.0401 | 11 | 7.9 |
| 46 | Ost e | 11 | 7.9 |
| 47 | Aln g 1 | 10 | 7.2 |
| 48 | Api m 1 | 10 | 7.2 |
| 49 | Der p 21 | 10 | 7.2 |
| 50 | Ory c 3 | 10 | 7.2 |
| 51 | Par j 2 | 10 | 7.2 |
| 52 | Fel d 4 | 9 | 6.5 |
| 53 | Mal d 1 | 9 | 6.5 |
| 54 | Pol d 5 | 9 | 6.5 |
| 55 | Api m | 8 | 5.8 |
| 56 | Ara h 9 | 8 | 5.8 |
| 57 | Der f 1 | 8 | 5.8 |
| 58 | Loc m | 8 | 5.8 |
| 59 | Api m 10 | 7 | 5.0 |
| 60 | Ara h 8 | 7 | 5.0 |
| 61 | Cor a_pollen | 7 | 5.0 |
| 62 | Der p 7 | 7 | 5.0 |
| 63 | Gly d 2 | 7 | 5.0 |
| 64 | Lol spp. | 7 | 5.0 |
| 65 | Mala s 6 | 7 | 5.0 |
| 66 | Vit v 1 | 7 | 5.0 |
| 67 | Api g 2 | 6 | 4.3 |
| 68 | Mus m 1 | 6 | 4.3 |
| 69 | Ory_meat | 6 | 4.3 |
| 70 | Pol d | 6 | 4.3 |
| 71 | Rat n | 6 | 4.3 |
| 72 | Ten m | 6 | 4.3 |
| 73 | Cup a 1 | 5 | 3.6 |
| 74 | Equ c 1 | 5 | 3.6 |
| 75 | Equ c_meat | 5 | 3.6 |
| 76 | Gly m 4 | 5 | 3.6 |
| 77 | Hom s LF | 5 | 3.6 |
| 78 | Lep d 2 | 5 | 3.6 |
| 79 | Pru p 3 | 5 | 3.6 |
| 80 | Sola l 6 | 5 | 3.6 |
| 81 | Ves v | 5 | 3.6 |
| 82 | Zea m | 5 | 3.6 |
| 83 | Zea m 14 | 5 | 3.6 |
| 84 | Aca s | 4 | 2.9 |
| 85 | Act d 10 | 4 | 2.9 |
| 86 | Ail a | 4 | 2.9 |
| 87 | Api g 6 | 4 | 2.9 |
| 88 | Bla g 2 | 4 | 2.9 |
| 89 | Bos d 6 | 4 | 2.9 |
| 90 | Cap h_milk | 4 | 2.9 |
| 91 | Dol spp | 4 | 2.9 |
| 92 | Fel d 2 | 4 | 2.9 |
| 93 | Mal d 3 | 4 | 2.9 |
| 94 | Ole e 9 | 4 | 2.9 |
| 95 | Pla a 2 | 4 | 2.9 |
| 96 | Pla a 3 | 4 | 2.9 |
| 97 | Ses i 1 | 4 | 2.9 |
| 98 | Tyr p | 4 | 2.9 |
| Prior to cluster analysis, allergens were filtered based on their prevalence within the analytical cohort, and were retained if present in at least 4 of the 139 sensitized patients (n ≥ 4). After filtering, 98 of 295 allergens were retained for analysis. | | | |

**Table S2.** **Pairwise comparisons between the five exploratory clusters, based on FDR-adjusted p-values.**

| **Comparison** | **AR** | **Asthma** | **AD** | **OAS** | **OAS through PR-10** | **OAS through Profilins** | **OAS through nsLTP** |
| --- | --- | --- | --- | --- | --- | --- | --- |
| **C1 (n=42) vs C2 (n=24)** | **0.007** | 0.9123 | 0.6285 | 1 | 1 | 1 | 1 |
| **C1 (n=42) vs C3 (n=47)** | **< 0.001** | 0.1995 | 0.269 | 1 | 1 | 1 | 1 |
| **C1 (n=42) vs C4 (n=10)** | 0.1526 | 0.269 | 1 | 0.1526 | 1 | 0.0756 | 0.1995 |
| **C1 (n=42) vs C5 (n=16)** | 0.063 | 0.4252 | 1 | 0.2634 | 0.1526 | 1 | 1 |
| **C2 (n=24) vs C3 (n=47)** | 1 | 0.9123 | 1 | 1 | 1 | 1 | 1 |
| **C2 (n=24) vs C4 (n=10)** | 1 | 0.9123 | 0.5053 | 0.269 | 1 | 0.1526 | 0.269 |
| **C2 (n=24) vs C5 (n=16)** | 1 | 1 | 0.9123 | 0.6608 | 0.2634 | 1 | 1 |
| **C3 (n=47) vs C4 (n=10)** | 1 | 1 | 0.2803 | 0.2634 | 1 | 0.0718 | 0.269 |
| **C3 (n=47) vs C5 (n=16)** | 1 | 1 | 0.4461 | 0.4461 | 0.1526 | 1 | 1 |
| **C4 (n=10) vs C5 (n=16)** | 1 | 1 | 1 | 1 | 0.6312 | 0.2488 | 0.4215 |
| Cells show FDR-adjusted p-values (Benjamini-Hochberg method) from pairwise Fisher's exact tests across all 70 comparisons (7 variables x 10 cluster pairs). Color intensity reflects significance level: white = p >= 0.05 (not significant); light red = 0.01 <= p < 0.05; medium red = 0.001 <= p < 0.01; dark red = p < 0.001. Non-significant pairwise comparisons involving small clusters (C4 n=10, C5 n=16) should be interpreted with caution given limited statistical power. AR: allergic rhinitis; AD: atopic dermatitis; OAS: oral allergy syndrome; PR-10: pathogenesis-related protein 10; nsLTP: non-specific lipid transfer protein. | | | | | | | |

**Table S3. Molecular sensitization profiles across the five exploratory clusters.**

| **Allergen** | **Overall n (%)** | **C1 (n=42)** | **C2 (n=24)** | **C3 (n=47)** | **C4 (n=10)** | **C5 (n=16)** |
| --- | --- | --- | --- | --- | --- | --- |
| **Act d 10** | 4 (2.9%) | 0% (p = 0.7262) | 0% (p = 1) | 0% (p = 0.7262) | **40% (p < 0.001)** | 0% (p = 1) |
| **Aln g 1** | 10 (7.2%) | 0% (p = 0.1488) | 4.2% (p = 1) | 2.1% (p = 0.5048) | **70% (p < 0.001)** | 6.2% (p = 1) |
| **Amb a** | 64 (46%) | **2.4% (p < 0.001)** | 54.2% (p = 0.8976) | **63.8% (p = 0.0262)** | 50% (p = 1) | **93.8% (p = 0.0013)** |
| **Amb a 1** | 66 (47.5%) | **4.8% (p < 0.001)** | 50% (p = 1) | **66% (p = 0.0182)** | 50% (p = 1) | **100% (p < 0.001)** |
| **Api g 2** | 6 (4.3%) | 0% (p = 0.5315) | 0% (p = 0.9301) | 4.3% (p = 1) | **30% (p = 0.0316)** | 6.2% (p = 0.9301) |
| **Api g 6** | 4 (2.9%) | 2.4% (p = 1) | 0% (p = 1) | 0% (p = 0.7262) | **30% (p = 0.01)** | 0% (p = 1) |
| **Api m** | 8 (5.8%) | 2.4% (p = 0.823) | **20.8% (p = 0.0279)** | 0% (p = 0.2182) | 10% (p = 0.8478) | 6.2% (p = 1) |
| **Ara h 8** | 7 (5%) | 0% (p = 0.3523) | 0% (p = 0.9301) | 0% (p = 0.3478) | **70% (p < 0.001)** | 0% (p = 1) |
| **Bet v 1** | 12 (8.6%) | 0% (p = 0.0914) | 8.3% (p = 1) | 6.4% (p = 1) | **70% (p < 0.001)** | 0% (p = 0.7699) |
| **Bet v 2** | 14 (10.1%) | **0% (p = 0.036)** | 0% (p = 0.4109) | **0% (p = 0.0193)** | 10% (p = 1) | **81.2% (p < 0.001)** |
| **Cor a 1.0103** | 13 (9.4%) | 2.4% (p = 0.3617) | 8.3% (p = 1) | 6.4% (p = 0.9301) | **60% (p < 0.001)** | 6.2% (p = 1) |
| **Cor a 1.0401** | 11 (7.9%) | 0% (p = 0.1543) | 4.2% (p = 0.9799) | 6.4% (p = 1) | **70% (p < 0.001)** | 0% (p = 0.77) |
| **Cor a_pollen** | 7 (5%) | 0% (p = 0.3523) | 8.3% (p = 0.7566) | 0% (p = 0.3478) | **50% (p < 0.001)** | 0% (p = 1) |
| **Cry j 1** | 37 (26.6%) | **7.1% (p = 0.0044)** | 33.3% (p = 0.8446) | 31.9% (p = 0.7262) | 10% (p = 0.7262) | **62.5% (p = 0.0131)** |
| **Cuc m 2** | 18 (12.9%) | **0% (p = 0.0138)** | 4.2% (p = 0.7262) | **2.1% (p = 0.0381)** | 20% (p = 0.938) | **87.5% (p < 0.001)** |
| **Cyn d** | 36 (25.9%) | **2.4% (p < 0.001)** | **95.8% (p < 0.001)** | **8.5% (p = 0.0086)** | 30% (p = 1) | 31.2% (p = 0.9301) |
| **Cyn d 1** | 39 (28.1%) | **4.8% (p < 0.001)** | **100% (p < 0.001)** | **10.6% (p = 0.0107)** | 30% (p = 1) | 31.2% (p = 1) |
| **Der f 2** | 26 (18.7%) | **0% (p < 0.001)** | 25% (p = 0.7957) | **40.4% (p < 0.001)** | 0% (p = 0.5764) | 6.2% (p = 0.7262) |
| **Der p 1** | 26 (18.7%) | **2.4% (p = 0.0071)** | 29.2% (p = 0.4928) | **34% (p = 0.0177)** | 0% (p = 0.5764) | 12.5% (p = 1) |
| **Der p 2** | 25 (18%) | **0% (p = 0.0013)** | 25% (p = 0.7905) | **38.3% (p < 0.001)** | 0% (p = 0.5764) | 6.2% (p = 0.7262) |
| **Der p 21** | 10 (7.2%) | 0% (p = 0.1488) | 4.2% (p = 1) | **17% (p = 0.0197)** | 0% (p = 1) | 6.2% (p = 1) |
| **Der p 23** | 20 (14.4%) | **2.4% (p = 0.0431)** | 25% (p = 0.3783) | 23.4% (p = 0.1774) | 0% (p = 0.7699) | 12.5% (p = 1) |
| **Der p 5** | 14 (10.1%) | 4.8% (p = 0.617) | 4.2% (p = 0.8478) | **21.3% (p = 0.0318)** | 0% (p = 0.9301) | 6.2% (p = 1) |
| **Der p 7** | 7 (5%) | 0% (p = 0.3523) | 4.2% (p = 1) | **12.8% (p = 0.038)** | 0% (p = 1) | 0% (p = 1) |
| **Fag s 1** | 13 (9.4%) | 4.8% (p = 0.7516) | 4.2% (p = 0.8499) | 6.4% (p = 0.9301) | **70% (p < 0.001)** | 0% (p = 0.77) |
| **Fel d 1** | 49 (35.3%) | **14.3% (p = 0.0086)** | 37.5% (p = 1) | **51.1% (p = 0.0478)** | 40% (p = 1) | 37.5% (p = 1) |
| **Fel d 7** | 15 (10.8%) | 2.4% (p = 0.17) | **29.2% (p = 0.0316)** | 12.8% (p = 0.9301) | 0% (p = 0.9301) | 6.2% (p = 1) |
| **Fra a 1+3** | 17 (12.2%) | 9.5% (p = 0.9301) | 4.2% (p = 0.7262) | 2.1% (p = 0.0644) | **100% (p < 0.001)** | 6.2% (p = 0.9799) |
| **Fra e** | 12 (8.6%) | 2.4% (p = 0.3581) | **25% (p = 0.0394)** | 6.4% (p = 1) | 10% (p = 1) | 6.2% (p = 1) |
| **Fra e 1** | 14 (10.1%) | 7.1% (p = 0.9301) | **29.2% (p = 0.0213)** | 6.4% (p = 0.7905) | 10% (p = 1) | 0% (p = 0.7748) |
| **Gly m 4** | 5 (3.6%) | 0% (p = 0.7262) | 0% (p = 0.9301) | 2.1% (p = 0.9692) | **40% (p = 0.0013)** | 0% (p = 1) |
| **Hev b 8** | 16 (11.5%) | **0% (p = 0.0209)** | 0% (p = 0.2968) | 2.1% (p = 0.0644) | 20% (p = 0.7262) | **81.2% (p < 0.001)** |
| **Jug r_pollen** | 12 (8.6%) | 2.4% (p = 0.3581) | 0% (p = 0.4097) | 4.3% (p = 0.7423) | 10% (p = 1) | **50% (p < 0.001)** |
| **Lol p 1** | 47 (33.8%) | **0% (p < 0.001)** | **100% (p < 0.001)** | 25.5% (p = 0.5416) | 40% (p = 1) | 43.8% (p = 0.7997) |
| **Mal d 1** | 9 (6.5%) | 0% (p = 0.2391) | 8.3% (p = 0.9692) | 2.1% (p = 0.7143) | **60% (p < 0.001)** | 0% (p = 0.9301) |
| **Mal d 3** | 4 (2.9%) | 0% (p = 0.7262) | 0% (p = 1) | 2.1% (p = 1) | **30% (p = 0.01)** | 0% (p = 1) |
| **Mer a 1** | 21 (15.1%) | **2.4% (p = 0.0282)** | 4.2% (p = 0.4043) | **2.1% (p = 0.0158)** | 20% (p = 0.9673) | **100% (p < 0.001)** |
| **Ole e 1** | 15 (10.8%) | 4.8% (p = 0.6235) | **29.2% (p = 0.0316)** | 6.4% (p = 0.7905) | 20% (p = 0.7262) | 6.2% (p = 1) |
| **Pas n** | 21 (15.1%) | **0% (p = 0.0044)** | **45.8% (p = 0.0013)** | 10.6% (p = 0.7381) | 10% (p = 1) | 25% (p = 0.7022) |
| **Phl p 1** | 46 (33.1%) | **0% (p < 0.001)** | **100% (p < 0.001)** | 23.4% (p = 0.3434) | 40% (p = 1) | 43.8% (p = 0.7997) |
| **Phl p 12** | 19 (13.7%) | **0% (p = 0.008)** | 4.2% (p = 0.5636) | **2.1% (p = 0.0245)** | 20% (p = 0.9482) | **93.8% (p < 0.001)** |
| **Phl p 2** | 17 (12.2%) | **0% (p = 0.0144)** | **41.7% (p < 0.001)** | **0% (p = 0.0071)** | 40% (p = 0.1035) | 18.8% (p = 0.8057) |
| **Phl p 5.0101** | 21 (15.1%) | **0% (p = 0.0044)** | **37.5% (p = 0.0187)** | 12.8% (p = 1) | 0% (p = 0.7699) | 37.5% (p = 0.0887) |
| **Phl p 6** | 15 (10.8%) | **0% (p = 0.0346)** | 25% (p = 0.1167) | 10.6% (p = 1) | 0% (p = 0.9301) | 25% (p = 0.2968) |
| **Pho d 2** | 20 (14.4%) | **0% (p = 0.0053)** | 4.2% (p = 0.5636) | **2.1% (p = 0.0163)** | 20% (p = 0.9562) | **100% (p < 0.001)** |
| **Pla a 3** | 4 (2.9%) | 0% (p = 0.7262) | 0% (p = 1) | 2.1% (p = 1) | **30% (p = 0.01)** | 0% (p = 1) |
| **Pru p 3** | 5 (3.6%) | 0% (p = 0.7262) | 4.2% (p = 1) | 2.1% (p = 0.9692) | **30% (p = 0.0191)** | 0% (p = 1) |
| **Sec c_pollen** | 29 (20.9%) | **2.4% (p = 0.0024)** | **50% (p = 0.0044)** | 19.1% (p = 1) | 10% (p = 0.9799) | 37.5% (p = 0.3523) |
| **Sola l 6** | 5 (3.6%) | 2.4% (p = 1) | 0% (p = 0.9301) | 0% (p = 0.5048) | **40% (p = 0.0013)** | 0% (p = 1) |
| **Ves v 5** | 14 (10.1%) | 21.4% (p = 0.061) | 12.5% (p = 0.9933) | **0% (p = 0.0193)** | 0% (p = 0.9301) | 12.5% (p = 0.9692) |
| **Vit v 1** | 7 (5%) | 0% (p = 0.3523) | 0% (p = 0.9301) | 2.1% (p = 0.8071) | **40% (p = 0.0044)** | 12.5% (p = 0.5416) |
| **Zea m** | 5 (3.6%) | 0% (p = 0.7262) | 0% (p = 0.9301) | 2.1% (p = 0.9692) | **40% (p = 0.0013)** | 0% (p = 1) |
| **Zea m 14** | 5 (3.6%) | 0% (p = 0.7262) | 0% (p = 0.9301) | 0% (p = 0.5048) | **50% (p < 0.001)** | 0% (p = 1) |
| Sensitization prevalence (%) within each cluster. Colored cells indicate statistically significant enrichment (prevalence > 0%, one-vs-rest Fisher's exact test, Benjamini-Hochberg FDR correction across 490 tests: 98 allergens x 5 clusters; significance threshold p < 0.05). Bold black text with p-value indicates significant depletion (prevalence = 0%, p < 0.05). Allergens sorted alphabetically. Cell color coding is consistent with cluster colors in Figures 2-4. C1: Pauci-sensitized, asthma-predominant; C2: Grass pollen-dominant; C3: Polysensitized; C4: Multi-panallergen (PR-10/nsLTP); C5: Ragweed and profilin-dominant. *Abbreviations: sIgE: specific immunoglobulin E; nsLTP: non-specific lipid transfer protein; PR-10: pathogenesis-related protein 10.* | | | | | | |
